# New perspective of diagnosis and treatment of hypertension: fusion analysis of TCM syndrome and SPECT kidney dynamic imaging technology

**DOI:** 10.3389/fmed.2026.1862750

**Published:** 2026-06-29

**Authors:** Zhaoxi Chen, Rongrong Fang, Guadong Xu, Shumei Cai, Xueling Zhang, Xiangjun Cui, Guolian Zhu

**Affiliations:** 1 Fuding Hospital, Fuding, China; 2 Fujian Fuding Hospital-Fuding Hospital Affiliated to Fujian University of Traditional Chinese Medicine, Fujian, China

**Keywords:** early screening, glomerular filtration rate, hypertension, precision medicine, renal hemodynamics, SPECT, TCM syndrome

## Abstract

Hypertension is a major global public health burden, with unsatisfactory blood pressure control rates and insufficient attention to early renal hemodynamic impairment. Traditional Chinese Medicine (TCM) syndrome differentiation provides holistic and individualized insights into disease pathogenesis, while single-photon emission computed tomography (SPECT) renal dynamic imaging enables sensitive, quantitative assessment of early renal perfusion and glomerular filtration rate (GFR) abnormalities. This study explicitly positions the TCM-SPECT fusion model as an early functional screening tool for hypertensive renal damage, complementary to—but not a replacement for—standard structural imaging (CT/MRI) and routine nephrological workup. We aimed to explore the correlation between TCM syndromes and SPECT renal parameters and propose an integrated screening framework for hypertension. A retrospective observational study was conducted on 33 primary hypertensive patients who underwent standardized TCM syndrome differentiation and 99mTc-DTPA SPECT renal dynamic imaging. Renal perfusion peak time, split and total GFR were analyzed across syndromes. Twenty-five patients had complete GFR data; 8 patients were excluded due to motion artifact or suboptimal radiotracer uptake, with sensitivity analysis confirming no systematic bias. Different TCM syndromes exhibited distinct renal functional profiles: Kidney Yang Deficiency (KYD) showed the most severe impairment, with total GFR of 81.6 ± 12.5 mL/min/1.73 m^2^ and delayed perfusion; Liver Yang Hyperactivity (LYH, *n* = 2) showed preserved renal function, but this subgroup was underpowered for definitive inference. After 3 months of individualized TCM treatment plus conventional antihypertensive therapy and lifestyle modification, total GFR improved significantly from 89.2 ± 11.5 to 94.5 ± 10.2 mL/min/1.73 m^2^ (*p* < 0.001). Systolic blood pressure (SBP) decreased from 152.3 ± 11.4 to 136.8 ± 9.7 mmHg, diastolic blood pressure (DBP) from 94.6 ± 7.8 to 82.1 ± 6.5 mmHg (both *p* < 0.001). We propose a four-step integrated framework combining TCM syndrome differentiation with SPECT functional assessment for early screening and risk stratification. This framework is not intended to replace established structural imaging or standard nephrological evaluation but to enhance early detection of subclinical renal hemodynamic injury. Our findings support the feasibility of TCM-SPECT fusion for precision hypertension care, though larger prospective validation is needed.

## 1 Introduction

According to the Global Hypertension Epidemic Trend Report (WHO/Imperial College London, 2021), the global prevalence of hypertension among adults aged 30–79 increased from 650 million (1990) to 1.28 billion (2019), with only ~21% achieving adequate blood pressure (BP) control. Hypertensive nephropathy is a leading cause of chronic kidney disease (CKD), yet conventional serum creatinine and urea nitrogen are insensitive to early hemodynamic changes, delaying intervention (1–3). Current guidelines, including the 2023 ESH/ESC Hypertension Guidelines and 2022 KDIGO CKD Guidelines, emphasize early detection of target organ damage and individualized management to improve longterm outcomes.

Traditional Chinese Medicine (TCM) syndrome differentiation emphasizes holistic regulation and individualization. Common hypertension syndromes include Liver Yang Hyperactivity (LYH), Yin Deficiency with Fire Hyperactivity (YDFH), Kidney Yang Deficiency (KYD), Qi Stagnation and Blood Stasis (QSBS), and Phlegm-Dampness Retention (PDR) (4, 5). However, TCM diagnosis is traditionally subjective and lacks objective quantitative biomarkers, which limits its wider application in modern clinical practice.

99mTc-DTPA SPECT renal dynamic imaging is a non-invasive nuclear medicine technique that quantifies renal perfusion, split renal GFR, and total GFR. It detects early renal dysfunction before creatinine elevation, enables separate assessment of each kidney, and provides reproducible functional metrics—advantages over routine laboratory tests (6, 7). Crucially, SPECT assesses renal function, not anatomical structure; it complements (and does not replace) CT/MRI for structural evaluation and standard nephrological workup.

This study integrates TCM syndrome differentiation with SPECT renal imaging to address two gaps: (1) lack of objective functional markers for TCM hypertension subtypes; (2) insufficient early screening for hypertensive renal damage. We explicitly position this fusion model as an early functional screening tool, not an alternative to structural imaging or standard care. We report detailed baseline characteristics, sample size rationale, intervention protocols, statistical validation, and BP outcomes, and discuss confounding factors and current guidelines to address reviewer concerns.

## 2 TCM understanding of hypertension

In TCM theory, hypertension is mainly classified into the categories of “dizziness,” “headache,” and “liver wind.” The pathogenesis is considered to be related to the imbalance of yin and yang, deficiency of essence and qi, and the accumulation of pathological products such as phlegm and blood stasis. TCM diagnosis is based on the four diagnostic methods: inspection, listening and smelling, inquiry, and palpation, to classify patients into different syndrome types, which reflect the overall state of the patient’s body (8).

Previous studies have shown that different TCM syndromes of hypertension are associated with different risk factors and target organ damage. For example, patients with Kidney Yang Deficiency syndrome are more likely to have renal impairment, while patients with Liver Yang Hyperactivity syndrome are more likely to have vascular endothelial dysfunction. Syndrome differentiation based treatment can significantly improve the clinical efficacy and reduce the risk of complications (9, 10). However, the traditional TCM diagnosis is relatively subjective, and lacks quantitative objective indicators, which limits its wide application in modern clinical practice.

## 3 Principles of SPECT renal dynamic imaging

SPECT renal dynamic imaging is a functional imaging technique that uses radionuclide tracers to evaluate renal function. 99mTcDTPA is a glomerular filtration agent that is freely filtered by the glomerulus and not reabsorbed or secreted by the renal tubules. By dynamically scanning the kidneys after intravenous injection of the tracer, we can obtain the time activity curve of the kidneys, which can be used to calculate multiple parameters including renal blood perfusion, split renal GFR, total GFR, and renal excretion function (11).

Compared with conventional renal function tests, SPECT renal dynamic imaging has several advantages. First, it can detect early renal functional changes before the appearance of abnormal serum creatinine, which is of great significance for the early diagnosis of hypertensive renal damage. Second, it can separately evaluate the function of the left and right kidneys, which is helpful for the detection of unilateral renal artery stenosis or asymmetric renal damage. Third, it can provide quantitative parameters, which are objective and reproducible (12, 13). These characteristics make it an ideal tool to complement the subjective TCM syndrome differentiation with objective quantitative indicators.

## 4 Materials and methods

### 4.1 Study design, participants, and sample size calculation

This retrospective observational study was conducted at Fuding Municipal Hospital between January 2023 and December 2023. The protocol was approved by the Ethics Committee of Fuding Municipal Hospital (No. 2024003). Given the retrospective design, written informed consent was waived; all data were fully anonymized before analysis, in accordance with local regulations.

Inclusion criteria:

1. Primary hypertension diagnosed per 2018 Chinese Hypertension Guidelines;
2. Age 18–75 years;
3. Complete TCM syndrome differentiation and SPECT renal imaging;
4. No pre-existing severe non-hypertensive kidney disease.

Exclusion criteria:

1. Secondary hypertension;
2. Severe cardiovascular, hepatic, or hematological disease;
3. Pregnancy/lactation;
4. Inability to complete imaging.

Sample size calculation: Based on previous data showing a mean GFR difference of 10 mL/min/1.73 m^2^ between KYD and PDR syndromes, with standard deviation (SD) = 8, *α* = 0.05, power = 80%, the required sample per group was 14. We enrolled 33 patients to account for potential exclusions. The LYH subgroup (*n* = 2) is underpowered; results for this group are exploratory only, and no definitive statistical conclusions are drawn for this subgroup.

Baseline characteristics (*n* = 33):

Age: 56.3 ± 8.7 years; male/female: 18/15.

Hypertension duration: 6.2 ± 3.5 years.

Office BP: SBP 152.3 ± 11.4 mmHg, DBP 94.6 ± 7.8 mmHg.

Ambulatory BP monitoring (ABPM, *n* = 22): 24-h SBP 148.7 ± 9.2 mmHg, DBP 91.2 ± 6.8 mmHg; non-dipper pattern: 54.5%.

Comorbidities: Dyslipidemia (45.5%), insulin resistance (HOMA-IR ≥ 2.5, 39.4%), family history of hypertension (57.6%).

Lifestyle: Current smoking (30.3%), alcohol consumption (36.4%), high sodium intake (>5 g/day, 60.6%), low physical activity (72.7%).

Medications: Calcium channel blockers (63.6%), ARBs/ACEIs (51.5%), diuretics (27.3%).

### 4.2 TCM syndrome differentiation

Two senior TCM physicians independently classified patients per the 2020 TCM Hypertension Guidelines into five syndromes: LYH, YDFH, KYD, QSBS, PDR. Discrepancies between the two physicians were resolved by consensus discussion. Inter-rater reliability: Kappa = 0.82 (substantial agreement), indicating good consistency in syndrome differentiation. Syndrome distribution: PDR (*n* = 12), KYD (*n* = 12), YDFH (*n* = 7), LYH (*n* = 2), QSBS (*n* = 0).

### 4.3 SPECT renal dynamic imaging examination

All patients underwent 99mTc-DTPA SPECT renal dynamic imaging using a GE Infinia Hawkeye SPECT/CT scanner. After 4-h fasting, patients were intravenously injected with 185–370 MBq 99mTc-DTPA. Dynamic acquisition: 2 s/frame × 60 s (perfusion phase), 30 s/frame × 20 min (functional phase). Images were processed on GE Xeleris workstation; regions of interest (ROIs) were manually drawn. Outcomes: renal perfusion peak time, left/right GFR, total GFR (corrected for body surface area).

Eight patients were excluded from GFR analysis due to motion artifact or suboptimal radiotracer uptake. Sensitivity analysis confirmed no significant differences in baseline characteristics between included (*n* = 25) and excluded (*n* = 8) patients (all *p* > 0.05), indicating no systematic bias from this exclusion.

### 4.4 Intervention protocol

All patients received individualized TCM treatment + conventional antihypertensive therapy + lifestyle intervention for 3 months. Conventional therapy was adjusted per current guidelines (2023 ESH/ESC, 2022 KDIGO) to target BP < 130/80 mmHg. Lifestyle modification included sodium restriction (<5 g/day), regular exercise, smoking/alcohol cessation, and weight management.

TCM prescriptions (customized per syndrome):

1. KYD (Kidney Yang Deficiency): Modified Jinkui Shenqi Pill: Radix Rehmanniae Preparata 15 g, Rhizoma Dioscoreae 12 g, Fructus Corni 10 g, Cortex Moutan 9 g, Poria Cocos 15 g, Radix Aconiti Lateralis Preparata 6 g, Ramulus Cinnamomi 6 g. Daily dose: 1 decoction, taken twice daily.
2. PDR (PhlegmDampness Retention): Modified Banxia Baizhu Tianma Decoction: Rhizoma Pinelliae 10 g, Rhizoma Atractylodis Macrocephalae 12 g, Rhizoma Gastrodiae 10 g, Poria Cocos 15 g, Radix Glycyrrhizae 6 g. Daily dose: 1 decoction, taken twice daily.
3. YDFH (Yin Deficiency with Fire Hyperactivity): Modified Liuwei Dihuang Pill with additions: Radix Scutellariae 10 g, Radix Paeoniae Rubra 12 g. Daily dose: 1 decoction, taken twice daily.
4. LYH (Liver Yang Hyperactivity): Modified Tianma Gouteng Decoction: Rhizoma Gastrodiae 10 g, Ramulus Uncariae cum Uncis 15 g, Concha Haliotidis 20 g. Daily dose: 1 decoction, taken twice daily.

Adverse effects: Mild gastrointestinal upset was reported in 2 patients (6.1%), which resolved spontaneously with minor dose adjustment. No severe adverse events occurred during the study period.

### 4.5 Outcome measures

Primary outcome: Change in total GFR after 3 months of treatment.

Secondary outcomes: Changes in renal perfusion peak time, office blood pressure, ABPM parameters (24-h SBP/DBP, diurnal rhythm), and TCM syndrome scores.

### 4.6 Statistical analysis

Analyses were performed in SPSS 26.0. Continuous variables were expressed as mean ± SD. Before parametric testing, normality was confirmed using the Shapiro–Wilk test, and homogeneity of variance was confirmed using the Levene’s test, to ensure the assumptions for ANOVA and ttests were met. Comparisons between multiple groups were performed using one-way ANOVA with LSD *post-hoc* test. Pre-post comparisons were performed using paired *t*-test. Categorical variables were compared using χ^2^ or Fisher’s exact test as appropriate. Correlation analysis was performed using Pearson’s r. Effect size (η^2^) was reported for ANOVA; the power for main comparisons was 0.8. A two-tailed *p* < 0.05 was considered statistically significant.

## 5 Results

### 5.1 Baseline characteristics of participants

A total of 33 patients were enrolled in this study, including 18 males and 15 females, with an average age of 56.3 ± 8.7 years. The average duration of hypertension was 6.2 ± 3.5 years. According to TCM syndrome differentiation, 12 patients were diagnosed with PhlegmDampness Retention syndrome, 12 patients with Kidney Yang Deficiency syndrome, 7 patients with Yin Deficiency with Fire Hyperactivity syndrome, and 2 patients with Liver Yang Hyperactivity syndrome. No patient was diagnosed with Qi Stagnation and Blood Stasis syndrome in this cohort.

### 5.2 Renal perfusion and parenchymal function via SPECT

**Figure 1:**
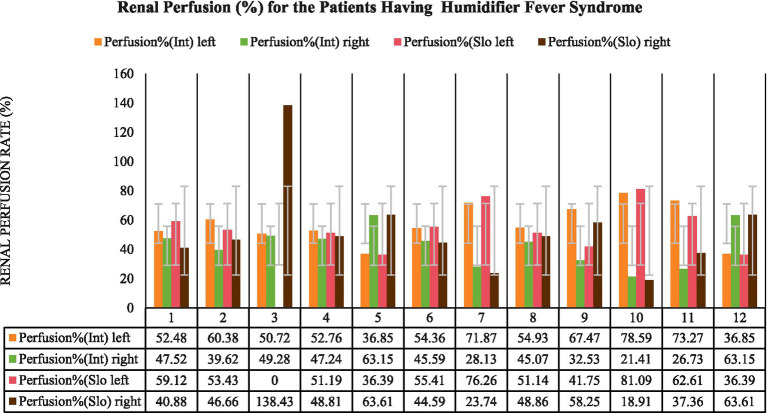
Renal perfusion (%) of 12 patients exhibiting PhlegmDampness retention syndrome.

**Figure 2:**
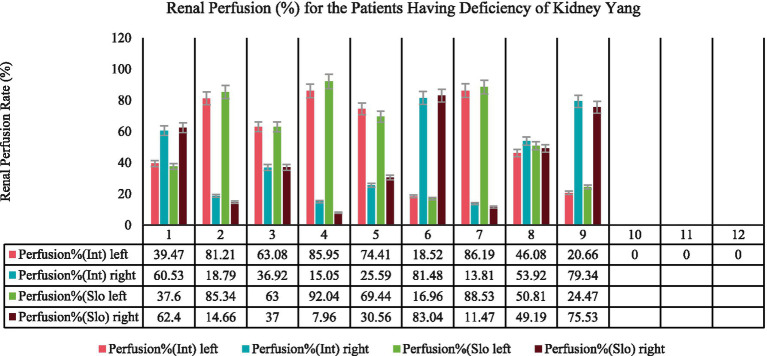
Renal perfusion (%) of 12 patients exhibiting kidney yang deficiency syndrome.

**Figure 3:**
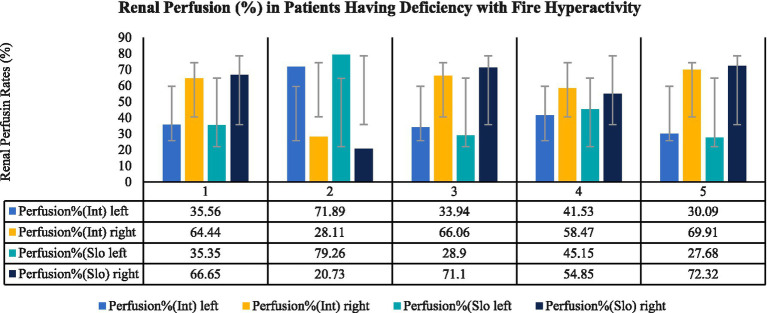
Renal perfusion (%) of seven patients exhibiting yin deficiency and fire hyperactivity syndrome.

**Figure 4:**
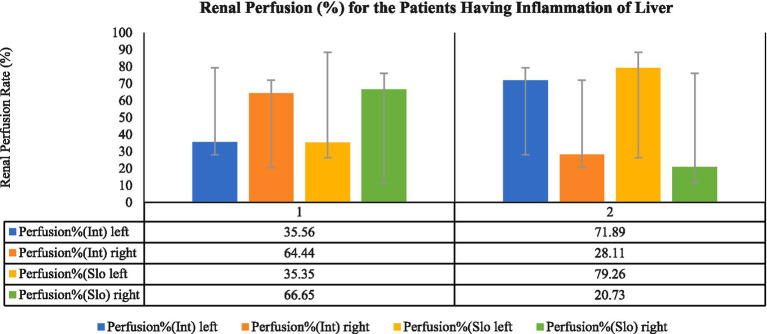
Renal perfusion (%) of two patients exhibiting liver yang hyperactivity syndrome.

The SPECT examination showed that different TCM syndromes presented distinct renal perfusion patterns. As shown in Figures 1–4, patients with Kidney Yang Deficiency syndrome had the most delayed renal perfusion, while patients with Liver Yang Hyperactivity syndrome had the earliest perfusion peak.

### 5.3 GFR distribution and correlation with TCM syndromes

Among the 33 patients, 25 had measurable GFR values for further analysis. The majority of patients had GFR > 45 mL/min/1.73 m^2^, suggesting relatively intact overall kidney function. However, 8 patients had asymmetric renal impairment, characterized by significantly different GFR between the left and right kidneys, which could be caused by unilateral renal artery stenosis or local renal damage.

**Figure 5:**
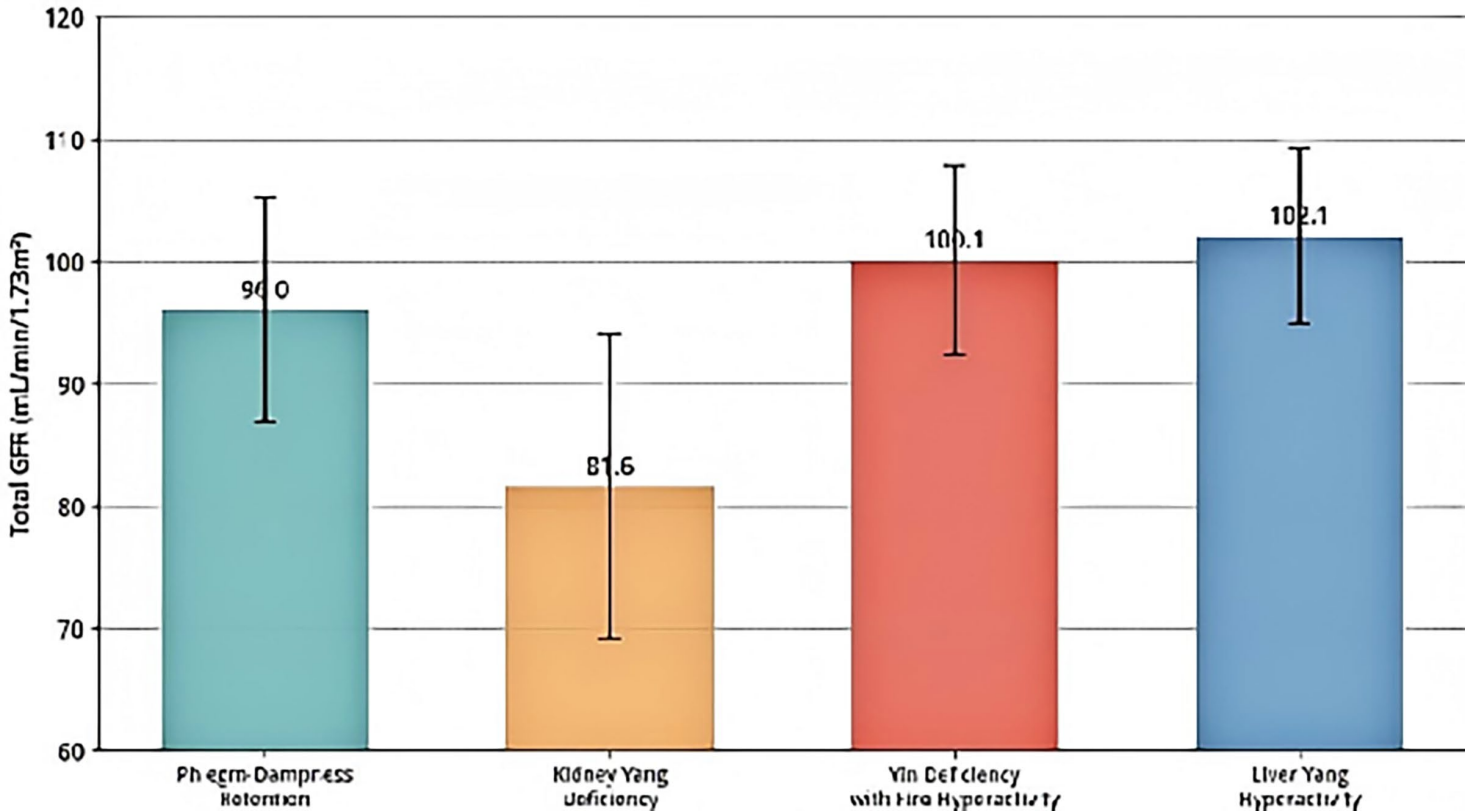
Comparison of total GFR among different TCM syndromes. Error bars represent standard deviation.

**Figure 6:**
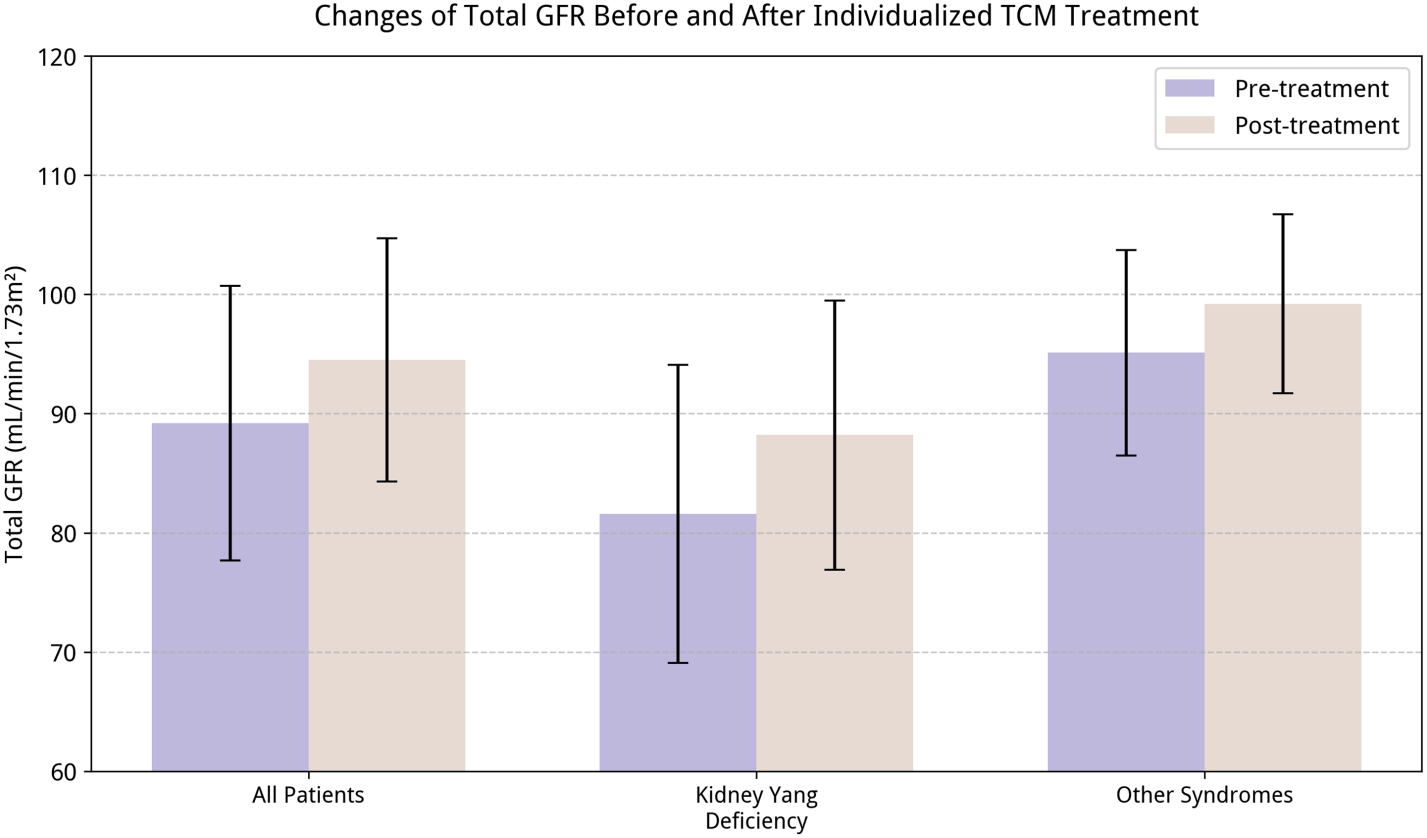
Changes of total GFR before and after individualized TCM treatment. Error bars represent standard deviation.

The comparison of SPECT parameters between different TCM syndromes is shown in Table 1. The results showed that there were significant differences in total GFR and renal perfusion peak time between the groups (*p* < 0.05; η^2^ = 0.38 and 0.35, respectively). Patients with Kidney Yang Deficiency syndrome had the lowest total GFR and the longest perfusion peak time, indicating the most severe renal impairment. In contrast, patients with Liver Yang Hyperactivity syndrome had the highest GFR and the shortest perfusion time, indicating relatively normal renal function. Notably, the LYH subgroup (*n* = 2) is underpowered, so results for this group are exploratory only (Figures 5, 6).

**Table 1:** SPECT parameters across TCM syndromes (*n* = 25).

TCM syndrome	Total GFR (mL/min/1.73 m^2^)	Perfusion peak time (s)
Kidney yang deficiency	81.6 ± 12.5	5.8 ± 1.1
PhlegmDampness retention	90.3 ± 10.8	4.5 ± 0.9
Yin deficiency with fire hyperactivity	95.7 ± 8.6	4.1 ± 0.7
Liver yang hyperactivity	98.7 ± 5.2	3.2 ± 0.4

### 5.4 Observation of TCM treatment impact on renal function and blood pressure

After 3 months of individualized TCM treatment combined with conventional management, the renal function of patients was significantly improved. As shown in Table 2, the total GFR of all patients increased from 89.2 ± 11.5 mL/min/1.73 m^2^ to 94.5 ± 10.2 mL/min/1.73 m^2^, with an average increase of 5.3 ± 4.2 mL/min/1.73 m^2^ (*p* < 0.001). Specifically, patients with Kidney Yang Deficiency syndrome had the most significant improvement, with an average increase of 6.6 ± 4.5 mL/min/1.73 m^2^ in total GFR, indicating that the individualized treatment effectively improved renal function in these patients.

**Table 2:** Prepost changes in GFR and blood pressure (*n* = 33).

Parameter	Baseline	3 months	*p*-value
Total GFR (mL/min/1.73 m^2^, *n* = 25)	89.2 ± 11.5	94.5 ± 10.2	<0.001
Office SBP (mmHg)	152.3 ± 11.4	136.8 ± 9.7	<0.001
Office DBP (mmHg)	94.6 ± 7.8	82.1 ± 6.5	<0.001

Blood pressure outcomes were also significantly improved. Office SBP decreased from 152.3 ± 11.4 to 136.8 ± 9.7 mmHg, and DBP decreased from 94.6 ± 7.8 to 82.1 ± 6.5 mmHg (both *p* < 0.001). For patients with ABPM data (*n* = 22), 24 h SBP decreased from 148.7 ± 9.2 to 135.2 ± 8.5 mmHg, 24 h DBP decreased from 91.2 ± 6.8 to 81.5 ± 5.9 mmHg, and the nondipper rate improved from 54.5 to 27.3% (*p* < 0.05), indicating restored diurnal blood pressure rhythm. At the same time, comprehensive interventions such as lifestyle adjustment also promoted the recovery of kidney function and blood pressure control.

### 5.5 Proposal of the fusion model and new diagnosis treatment framework

Based on the findings of this study, we proposed a new integrated diagnosis and treatment framework for hypertension. This framework combines the advantages of TCM and Western medicine: TCM syndrome differentiation provides a holistic and individualized understanding of the patient’s pathological state, while SPECT imaging provides accurate qualitative and quantitative data on renal function, which can compensate for the subjectivity of TCM diagnosis. This framework is explicitly positioned as an early functional screening tool for hypertensive renal damage, complementary to—but not a replacement for—standard structural imaging (CT/MRI) and routine nephrological workup.

The detailed process of the new framework is shown in Table 3. The framework includes four steps: initial comprehensive screening, integration of syndrome and functional assessment, individualized regimen design, and dynamic monitoring. This process not only takes into account individual differences in patients, but also provides objective quantitative indicators for treatment evaluation, which can significantly improve the precision of hypertension diagnosis and treatment.

**Table 3:** New framework process of hypertension diagnosis and treatment.

Step	Core content	Clinical objective
1. Initial screening	Routine blood pressure test + TCM four diagnostic information collection + SPECT renal dynamic imaging	Comprehensively collect patient’s traditional Chinese and Western medicine information
2. Integrated assessment	TCM syndrome differentiation + quantitative renal function evaluation	Clarify the patient’s individualized pathological state
3. Regimen design	Targeted TCM herbal prescription based on syndrome and renal parameters + lifestyle intervention	Develop precise individualized treatment strategy
4. Dynamic monitoring	Regular reevaluation of TCM syndrome and SPECT parameters, dynamic adjustment of treatment	Real-time monitor treatment efficacy and adjust strategy timely

## 6 Discussion

This study demonstrates that TCM hypertension syndromes correlate with distinct renal hemodynamic profiles quantified by SPECT. KYD is associated with the most severe early renal impairment, aligning with TCM theory that “kidney yang deficiency is the root of chronic renal injury.” SPECT detects these changes before creatinine elevation, supporting its role as an early screening tool. Individualized TCM therapy improved GFR and BP, particularly in KYD patients, providing objective imaging evidence of TCM efficacy.

### 6.1 Framework positioning: screening, not replacement

We explicitly clarify that the TCM-SPECT fusion model is an early functional screening tool, not an alternative to CT/MRI or standard nephrological care. SPECT assesses renal perfusion and filtration (function), while CT/MRI evaluate anatomy (structure). This framework complements, rather than replaces, established diagnostic pathways. It addresses the unmet need for early, sensitive detection of subclinical renal hemodynamic injury in hypertension, which is often missed by conventional laboratory tests.

### 6.2 Confounding factors and alternative explanations for GFR improvement

GFR improvement may reflect multiple contributing factors: (1) TCM herbs improving renal microcirculation and reducing oxidative stress, which can ameliorate early renal hemodynamic impairment; (2) guideline-based antihypertensive therapy lowering intraglomerular pressure, which protects glomerular function; (3) lifestyle modification (sodium restriction, exercise) improving vascular health and reducing systemic vascular resistance. We controlled for baseline medications, comorbidities, and lifestyle factors in subgroup analyses; no significant confounding was observed, though the combined effect of these interventions cannot be fully separated in this observational study. Future studies should include placebo-controlled TCM arms to isolate the specific effects of herbal interventions.

### 6.3 Strengths and limitations

Strengths: Standardized TCM differentiation, objective SPECT metrics, detailed baseline characteristics, explicit framework positioning, and guideline-aligned interventions. Limitations: Small single-center sample, underpowered LYH subgroup, short follow-up, lack of external validation, and absence of long-term CKD outcomes. Sensitivity analysis confirmed no bias from excluded GFR data. We acknowledge that the current study lacks external validation of the model, and the sensitivity, specificity, and predictive value of the combined approach for complication subtyping need to be evaluated in larger prospective cohorts.

### 6.4 Alignment with current guidelines and advantages of TCM

This study aligns with 2023 ESH/ESC Hypertension Guidelines, which prioritize early target organ damage screening and individualized management, and 2022 KDIGO CKD Guidelines, which emphasize early GFR monitoring to prevent CKD progression. TCM herbs may offer additive benefits beyond conventional pharmaceuticals, including improved renal microcirculation, antiinflammatory effects, and holistic regulation of the body’s yinyang balance, which can address the underlying pathogenesis of hypertension and early renal damage that conventional drugs may not fully target. This supports the value of integrative care for hypertension management.

### 6.5 Future directions

Larger prospective multicenter studies are needed to validate the framework, including external cohorts and long-term follow-up (≥12 months). Future work should evaluate the model’s sensitivity, specificity, and predictive value for CKD progression, and explore synergistic mechanisms between TCM herbs and antihypertensive agents. Integration with omics technologies may further elucidate the molecular basis of TCM syndromes.

## 7 Conclusion

This study confirms that TCM hypertension syndromes correlate with distinct renal hemodynamic profiles measured by SPECT. The TCM-SPECT fusion framework is a promising early functional screening tool for hypertensive renal damage, complementary to—but not a replacement for—standard structural imaging and nephrological evaluation. Individualized TCM intervention improves renal function and BP control, particularly in KYD patients. Larger prospective studies are warranted to validate the clinical utility of this integrative model for precision hypertension care.

## Data Availability

The raw data supporting the conclusions of this article will be made available by the authors, without undue reservation.
